# Sperm collection and characteristics analysis of the critically endangered Chinese pangolin (*Manis pentadactyla*)

**DOI:** 10.1093/conphys/coae010

**Published:** 2024-07-01

**Authors:** Yongzheng Li, Yan Hua, Zuofu Xiang, Xuelin Xu, Sunxiya Zhang, Xianghe Wang, Fuyu An, Zhenyu Ren, Kai Wang

**Affiliations:** Guangdong Provincial Key Laboratory of Silviculture, Protection and Utilization, Guangdong Academy of Forestry, Guangzhou, 510520, PR China; College of Life Sciences and Technology, Central South University of Forestry and Technology, Changsha, 410004, PR China; Guangdong Provincial Key Laboratory of Silviculture, Protection and Utilization, Guangdong Academy of Forestry, Guangzhou, 510520, PR China; College of Forestry, Central South University of Forestry and Technology, Changsha, 410004, PR China; Guangdong Provincial Key Laboratory of Silviculture, Protection and Utilization, Guangdong Academy of Forestry, Guangzhou, 510520, PR China; Department of Design, Shanghai Jiao Tong University, Shanghai, 201100, PR China; Guangdong Provincial Key Laboratory of Silviculture, Protection and Utilization, Guangdong Academy of Forestry, Guangzhou, 510520, PR China; Guangdong Provincial Key Laboratory of Silviculture, Protection and Utilization, Guangdong Academy of Forestry, Guangzhou, 510520, PR China; Guangdong Provincial Key Laboratory of Silviculture, Protection and Utilization, Guangdong Academy of Forestry, Guangzhou, 510520, PR China; Guangdong Provincial Key Laboratory of Silviculture, Protection and Utilization, Guangdong Academy of Forestry, Guangzhou, 510520, PR China

**Keywords:** Chinese pangolin, sperm collection, sperm quality assessment, semen morphology

## Abstract

The Chinese pangolin (*Manis pentadactyla*) is a critically endangered species. However, there is a paucity of research on the male reproductive gamete biology of this species. The present study was the first to systematically analyse the sperm characterization of the Chinese pangolin, including semen collection, sperm morphometry and ultrastructure. The semen of five male Chinese pangolins was successfully collected using the electroejaculation method. CASA (computer-assisted sperm analysis) was used to assess semen quality and take images for sperm morphometric analysis. Scanning electron microscopy (SEM) and transmission electron microscopy (TEM) were used for sperm ultrastructure observation. The results showed that the semen of the Chinese pangolin was yellow to pale yellow in colour, viscous, with a fishy odour, and a slightly alkaline pH of between 7.7 and 7.9. The head defects were the main sperm defects; there were 13 kinds of head defects counted in this study. The total sperm length, head length, head width and tail length were 67.62 ± 0.21 μm, 10.47 ± 0.06 μm, 1.33 ± 0.006 μm and 57.16 ± 0.20 μm, respectively. SEM observed that the spermatozoa had a rod-shaped head with a distinct apical ridge, which was different from most mammals and similar to that in avians and reptiles. Interestingly, TEM found that the acrosome membrane of the Chinese pangolin had a double membrane structure rather than a multiple bi-lamellar membrane structure as reported by the previous study. Collectively, this study contributes to the development of artificial breeding efforts and assisted reproductive techniques for the Chinese pangolin, as well as providing technical support for research on germplasm conservation of this species.

## Teaser

Sperm characterization is an important part of reproductive biology and is particularly important for the conservation of endangered species in the wild. However, research on this aspect of the Chinese pangolin, an endangered species from mainland China, was extremely limited. Therefore, the present study fills the gap in this research area by first systematically providing the sperm collection methods and the characteristics of the spermatozoa of Chinese pangolin.

## Introduction

The Chinese pangolin (*Manis pentadactyla*) belongs to Mammalia, Pholidota, Manidae and Manis and is widely distributed in most areas south of the Yangtze River in China and the archipelagos of Hainan and Taiwan, with three subspecies: the South China subspecies (*M. p. aurita*), the Hainan subspecies (*M. p. pusilla*) and the Taiwan subspecies (*M. p. pentadactyla*) (Wu *et al.*, 2002; Wu *et al.*, 2020b; Fuwen *et al.*, 2021). The Chinese pangolin has been over-poached due to the huge demand for traditional medicines, health products, leather and decorations (Wang, 2021). In addition, due to human activities such as deforestation, agricultural growth and urbanization, the habitat of the Chinese pangolin has been greatly destroyed (Wu, 2004; Katuwal *et al.*, 2017; Acharya *et al.*, 2018). Together, these factors have led to a sharp decline in the wild population of the Chinese pangolin. As a result, the Chinese pangolin was listed as ‘critically endangered’ on the Red List of Threatened Species by the World Conservation Union (IUCN) (Challender *et al.*, 2019). With the gradual depletion of wild populations, captive breeding has inevitably become an important way to protect the pangolin from extinction. However, the Chinese pangolin still faces the problems of low reproductive efficiency and insufficient research on its reproductive biology. For example, parameters and characteristics are unclear in the sperm of the Chinese pangolin, which leads to many technical obstacles in captive breeding (Yang *et al.*, 2007; Chin *et al.*, 2012a; Hua *et al.*, 2015).

On the one hand, the pangolin has poor reproductive potential, with one cub per year and rarely two cubs (T-LON, 2008; Chin *et al.*, 2012a), resulting in the slow growth of its population in the wild. On the other hand, there have been rare reports of successful artificial breeding of the Chinese pangolin in the last century (Hua *et al.*, 2015; Wu *et al.*, 2016); for example, in 1965, a Chinese pangolin pup was unknowingly born and successfully reared at the Ueno Zoo (Masui, 1967). In 2006–2007, the Chinese pangolin successfully gave birth to three cubs in Taipei Zoo, one of which died after giving birth and one of which died from the mother’s lack of ability to breastfeed (Chin *et al.*, 2012b). And in 2011, the Pangolin Artificial Rescue and Conservation and Breeding Research Base (PRB-SCNU) of South China Normal University (SCNU) produced one individually developed male offspring (MP86), which was found dead (Wu *et al.*, 2016). As of 2016, only five cases of captive-bred Chinese pangolin hatchlings have been reported, of which three survived and two died (Wu *et al.*, 2016). Our research team has conducted captive breeding studies of the Chinese pangolin since 2019 and has successfully bred 10 offspring in the first generation and one offspring in the second generation (unpublished data). In addition, the Malay pangolin (*Manis javanica*), which is also distributed in southern China, has been successfully bred and raised to the third generation of offspring (Yan *et al.*, 2021).

Assisted reproductive technology (ART) is one of the most effective solutions to reproductive problems in livestock, poultry and wildlife (Holt and Lloyd, 2009; Blackburn, 2018). Assisted reproductive technologies have been widely used in farm animals, poultry, mammals and zoo breeding since the last century (Burrows and Quinn, 1935; Hansen, 2014; Alexandre *et al.*, 2016; Herrick, 2019), and semen collection and sperm evaluation, which is an important component of assisted reproductive technologies, has been widely used in wildlife, such as Asian elephants (*Elephas maximus*), Tasmanian devil (*Sarcophilus harrisii*), La Plata three-banded armadillo (*Tolypeutes matacus*), giant anteaters (*Myrmecophaga tridactyla*) and giant pandas (*Ailuropoda melanoleuca*) (Schmitt and Hildebrandt, 1998; Herrick *et al.*, 2002; Keeley *et al.*, 2012; Luba *et al.*, 2015, Martin-Wintle *et al.*, 2019). In addition, during 2018–2019, Reza Tarmizi *et al.* attempted semen collection using rectal massage, rectal electrical stimulation, and a combination of both in 15 male Malayan pangolins (*Manis javanica*), and semen quality was assessed (Tarmizi *et al.*, 2020b). In summary, assisted reproductive technology is expected to be an effective strategy to solve the reproduction problem of the Chinese pangolin, especially for the problems of poor estrus performance and sperm quality in pangolin, and to increase the reproduction rate in captivity. However, there is almost no research on semen collection in the Chinese pangolin and the features of its spermatozoa.

Therefore, this study explored semen collection methodology for the Chinese pangolin from the perspective of assisted reproduction in wildlife, evaluated the semen quality, and described its sperm micromorphology and ultrastructural characteristics. It lays the foundation for the storage of the semen of this species and the establishment of a gene bank. In addition, the technique can be used to screen out excellent breeding males with genetic value and provide technical support for the development of artificial insemination, subsequently increasing the population in *ex situ* conservation.

## Materials and Methods

### Ethics approval

All the animal experiment and sample collection procedures were approved by the Guangdong Academy of Forestry (00202023–15/2/2023), with administrative support and permission from the Guangdong Provincial Wildlife Rescue Monitoring Center (200023–1/3/2023).

### Animals and housing

The five male Chinese pangolins involved in this experiment were all in good condition, were disease-free, weighed between 3 kg and 5 kg, and originated from the Guangdong Wildlife Rescue Monitoring Center (see Table 1 for details). Prior to the semen collection procedure in April 2023, the five pangolins were kept in housing built for pangolins at the Guangdong Wildlife Rescue Center. All pangolins were housed in a single room, divided into an inner and outer house, with the inner house for rest and diet and the outer house for activities. The inner house (2.7 m in length, 2.15 m in width and 2.8 m in height) was made of a row of single rooms, and each single room contained a nesting box (0.65 m in length, 0.55 m in width, 0.45 m in height) for the pangolin to reside in, in which dry straw and a water basin were placed; the outer house (6 m in length and 2.7 m in width) was made of natural earth formed into an earthen mound planted with greenery in which the pangolins could make holes.

**Table 1:** Location, age, weight, origin and mating behaviour of the five Chinese pangolins

Pangolin-ID	Location	Age (years)	Weight (kg)	Origin	Mating behaviour
Z3	Heyuan, Guangdong	≥4	5.43	WR	Yes
Z7	Chiu Chow, Guangdong	≥4	5.84	WR	Yes
Z11	Shanwei, Guangdong	≥3	4.30	WR	Yes
Z15	Shaoguan, Guangdong	≥2	3.41	WR	Yes
Z2–1	Guangzhou, Guangdong	2	5.11	CB	Yes

CB (captive bread); WR (wild recuse).

### Semen collection

In this experiment, the electroejaculation (EEJ) method was used for semen collection. The electroejaculation method is a common, safe and effective method for semen collection, especially for wildlife applications, because it can be successfully performed under anaesthesia. It consists of controlled electrical stimulation of the ejaculatory reflex using a rectal probe coupled to a specific voltage-generating unit to apply a stimulus that induces ejaculation in the animal (Silva *et al.*, 2004).

**Figure 1:**
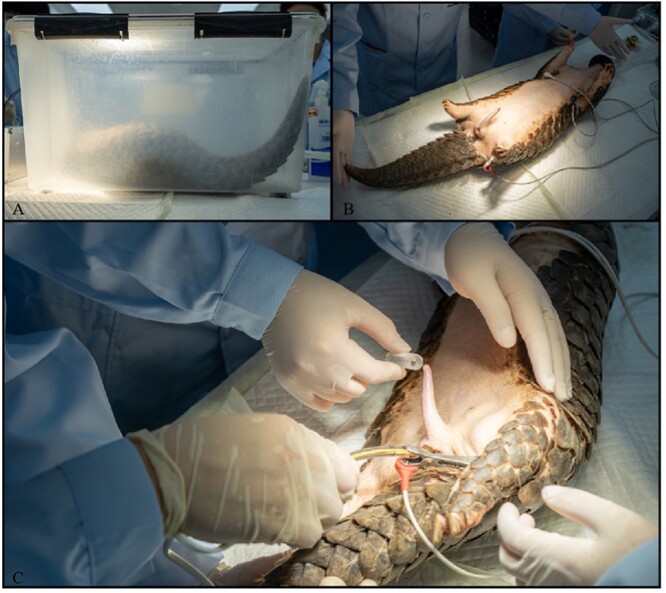
Chinese pangolin anaesthesia and semen collection. (**A**) Anaesthesia in an anaesthesia box; (**B**) anaesthesia mask and monitoring; (**C**) pangolin electrical stimulation for sperm collection.

Before semen collection was performed, the test animals were deprived of water and food for one day. Then, respiratory anaesthesia and semen collection were performed on the pangolin as follows: gas anaesthesia was performed on males with a 2% concentration of isoflurane (R510–22–10, Shenzhen RWD Life Science and Technology Co., Ltd, Shenzhen, China) (Fig. 1A and B) (An *et al.*, 2023). The pangolin was first placed into a transparent plastic box filled with anaesthesia gas, and after its response began to be delayed, it was quickly taken out and put into an anaesthesia mask while its heart rate was monitored. After the animal was stabilized, the abdomen and penis of the pangolin were squeezed to discharge as much urine as possible from the body to prevent contamination, and the perianal area, penis and prepuce were cleansed with physiological saline (0.9% NaCl). Sperm was collected using a three-electrode electroejaculator (MDW1, Chengdu Huazhi Kaiwu Technology Co., Ltd, Chengdu, Sichuan, China) with controlled current and voltage; the electrostimulator rod was 0.8 cm in diameter and 12 cm in length and was inserted into the rectum of male pangolins (Fig. 1C). In this experiment, initial rectal insertion depth was 3 cm. Continuous stimulation was applied for 5–10 seconds at 5-second intervals, with a starting voltage of 3 V, increasing sequentially in 0.25 V increments to a maximum voltage that did not exceed 7 V. The process was repeated and did not last longer than 10 minutes. The first segment of semen is thin and has impurities, which is not suitable for collection. The latter segment is a gushing thick semen, and when it is at this stage, the collection is successful. It should be emphasized that during the anaesthesia process, real-time heart rate detection should be carried out to prevent the animals from experiencing cardiac arrest after being stimulated to ensure the safety of the animals’ lives. Due to the characteristics of the animal itself and the consideration of animal ethics, semen collection was not repeated after the first collection from each male pangolin. If the collection failed, semen collection was repeated after a two-week interval.

### Semen evaluation

#### Computer-assisted sperm analysis (CASA)

With the development of technology in recent years, computer-assisted sperm analysis systems have been used in the assessment of sperm quality in domestic as well as wild animals (Broekhuijse *et al.*, 2012; Dogliero *et al.*, 2015; Basioura *et al.*, 2020, Schmidt *et al.*, 2021). The CASA system combines video microscopy with digital image capture and analysis for sperm identification and tracking. The system typically contains a frame store capable of collecting 20–30 consecutive video frames at a rate of 25–30 frames per second. It is important to note that because sperm may contain impurities, marked impurities need to be manually removed from the video before the system program processes the video frames to improve the accuracy of the detection. Therefore, in this study, a computer-assisted analysis system (ML-608JZIII, Nanning Song Jing Tianlun Biotechnology Co., Ltd, Nanning, Guangxi, China) was used to analyse the density, total motile spermatozoa (TMS, %), progressive motile sperm (PMS, %), straight line velocity (VSL, μm/s), curvilinear velocity (VCL, μm/s), average path velocity (VAP, μm/s), amplitude of lateral head displacement (ALH, μm), straightness (STR), linearity (LIN) and beat/cross-frequency (BCF, Hz) of the semen of the Chinese pangolin (Yu *et al.*, 2018). The morphology of the semen was determined by visual inspection and categorized as viscous, normal or thin. The collected semen was initially diluted with isothermal saline at a ratio of 1:3, and the diluted semen was slowly added to a four-chambered sperm counting plate (ML-CASA 20, Nanning Song Jing Tianlun Biotechnology Co., Ltd, Nanning, Guangxi, China) with a pool depth of 20 μm that had been prewarmed to an isothermal temperature and then tested for the various indices. The instrument parameters were set as follows: sperm dynamics analysis, 30 frames per second (HZ), 10× phase contrast objective (magnification: ×100) and at least five fields of view collected in each chamber; deformity rate, 20× phase contrast objective (magnification: ×200) and at least five fields of view collected in each chamber (the above parameters were based on the characteristics of Chinese pangolin semen and were verified and adjusted by several tests).

#### Semen pH

Semen pH was measured with high-precision pH indicator strips (pH 6.5–10, 109 543, Merck KGaA, Hessen, Germany).

#### Assessment of acrosome integrity and plasma membrane integrity

Giemsa staining is a commonly used method to detect the integrity of sperm acrosomes, which has the advantage of being simple and easy to observe (Watson, 1975). Therefore, in the present study, acrosome staining was performed using Wright-Giemsa staining solution (Changde Bikman Biotechnology Co., Ltd, Changde, Hunan, China), which is an improved version of the Giemsa stain, with the features of obvious colouring partition of the cellular structure, a short procedure, and the ability to be performed in batches. After staining, the cells were observed with a light microscope (oil microscope, magnification: ×1000; OLYMPUS CX21, Olympus Corporation, Tokyo, Japan). Light pink or light colour indicated the nucleus, and a dark purple or dark colour at the tip was considered to indicate an intact acrosome. If the tip was not stained or mauve in colour or the staining area was too large, the acrosome was considered to be incomplete. At least 200 spermatozoa were counted in five different fields of view for each sample.

The hypotonic swelling test (HOST) was used to evaluate the plasma membrane integrity of spermatozoa (Ejaz *et al.*, 2017). The HOST solution was a solution consisting of 100 ml of distilled water, 0.73 g of sodium citrate (YS175647, Beijing Solarbio Science & Technology Co., Ltd, Beijing, China), and 1.35 g of fructose (F8100, Beijing Solarbio Science & Technology Co., Ltd, Beijing, China) (osmotic pressure 190 mOsmol/kg) (Ahmed *et al.*, 2021). Due to the low volume of ejaculate in the Chinese pangolin, 10 μL of semen was mixed with 100 μL of HOST solution. Meanwhile, the Chinese pangolin is a low-temperature animal (average temperature 32.5°C, abdominal cavity temperature) and the testes are intra-abdominal (these data were obtained from unpublished clinical and feeding data). Therefore, the incubation temperature of spermatozoa was set to 32.5°C and incubated for 30 minutes. After incubation, the mixture was slowly added to a four-chambered sperm counting plate (ML-CASA 20, Nanning Song Jing Tianlun Biotechnology Co., Ltd, Nanning, Guangxi, China) with an isothermal pool depth of 20 μm and was evaluated by observation with a phase-contrast microscope (magnification: ×200; ML-CASA 20, Nanning Song Jing Tianlun Biotechnology Co., Ltd, Nanning, Guangxi, China). A curled tail indicated a complete sperm plasma membrane; an uncurled tail indicated an incomplete sperm plasma membrane. At least 200 spermatozoa were counted in five different fields of view and the number of intact and damaged plasma membranes was recorded separately.

### Sperm morphometry

The morphological description of Chinese pangolin spermatozoa in this study consisted of two parts: the first part was the measurement of sperm morphology and the morphological description of Chinese pangolin spermatozoa by ordinary optical microscopy, and the second part was the observation of the micromorphology and ultrastructure of Chinese pangolin spermatozoa using scanning electron microscopy (SEM) and transmission electron microscopy (TEM).

#### Sperm morphology analysis

Sperm morphology measurements, including head length, head width, tail length (including the middle part) and full length, were measured. No less than 200 spermatozoa per sample were measured using image analysis measurement software (Nanning Song Jing Tianlun Biotechnology Co., Ltd, Nanning, Guangxi, China). A phase contrast microscope (magnification: ×400; ML-CASA 20, Nanning Song Jing Tianlun Biotechnology Co., Ltd, Nanning, China) was used to analyse the morphology of the spermatozoa of the Chinese pangolin, mainly analysing the differences in the morphology of the head and the tail between normal spermatozoa and non-normal spermatozoa.

#### SEM

The reagents for this test were obtained from Sinopharm Chemical Reagent Co. (Shanghai, China). The collected fresh semen was centrifuged, and the precipitate was collected and immersed in PBS (0.1 M, without NaCl), rinsed several times, centrifuged to remove the supernatant, and then added to 4% glutaraldehyde electron microscopy fixative (P1127, Beijing Solarbio Science & Technology Co., Ltd, Beijing, China). The samples were precooled at 4°C, fixed at 4°C for 4 h or overnight, aspirated out of the fixative and immersed in PBS (0.1 M, without NaCl) 3–5 times for 15 min each time. The fixative was aspirated, and the samples were washed with PBS (0.1 M, NaCl-free) 3–5 times for 15 min each time, dehydrated with a series of graded alcohols (30%, 50%, 70%, 80%, 90%, 95%, 100%) for 15 min in each concentration, and then thoroughly dehydrated with 100% alcohol twice. Then, isoamyl acetate was added three times for 20 min each time, followed by drying (Downing Meisner *et al.*, 2005). After vacuum spray plating was completed, the samples were observed under a scanning electron microscope (HITACHI U8010, Hitachi Production Co., Ltd, Tokyo, Japan).

#### TEM

The reagents for this test were obtained from Sinopharm Chemical Reagent Co. (Shanghai, China). Fresh semen, collected by centrifugation, was added to 4% glutaraldehyde electron microscopy fixative (P1127, Beijing Solarbio Science & Technology Co., Ltd, Beijing, China) and stored at 4°C for at least 4 hours. Then, the samples were rinsed with 0.1 M phosphate buffer (pH 7.4) three times for 15 min each time. The samples were fixed in a mixture of 1% osmium acid and 0.1 M phosphate buffer (pH 7.4) for 2 hours at room temperature (20°C). Cells were then rinsed three times for 15 min in 0.1 M phosphate buffer (pH 7.2). Cells were subjected to an alcohol gradient (30%, 50%, 70%, 80%, 85%, 90% and 100% twice each) of upward dehydration for 15–20 min each time. The permeabilizing agent was acetone : epoxy resin (2 : 1), acetone : epoxy resin (1 : 1) and epoxy resin, in that order. Permeabilization was performed for 8–12 hours at 37°C in a temperature chamber. The permeabilized samples were placed into the embedding plate, added to the embedding agent (epoxy resin), and polymerized for 48 hours at 60°C in a warm box. Slices were sectioned at 80–100 nm using an ultrathin microtome (Leica EM UC7, Leica Mikrosysteme GmbH, Wetzlar, Germany) and stained with uranium–lead double staining (2% saturated aqueous uranium acetate and lead citrate) for 15 min at room temperature. The slices were dried overnight at room temperature (Cunha *et al.*, 2021), and the samples were analysed in a transmission electron microscope (TECNAI G 20 TWIN, FEI company, Hillsboro, Washington, Oregon, USA) for observation.

### Statistical analysis

The data were analysed using the statistical software IBM® SPSS® Statistics version 26 (IBM, Armonk, Westchester, New York, USA). The results are expressed as the mean ± SEM. The seminal parameter values for the five animals were analysed by *t* test. The seminal nonparametric data (head length and width, tail length, total sperm length) were subjected to one-way ANOVA followed by Dunn’s test for comparison of means. Significance was set at *P* < 0.05.

## Results

All male pangolins were in good physical condition throughout the test period, and all physiological indicators remained stable throughout the sperm collection process, with no adverse reactions after anaesthesia.

### Sperm collection and evaluation

In this experiment, semen was collected from five Chinese pangolins using the electroejaculation method. One male pangolin, number Z7, had semen contaminated with urine (data from the semen quality assessment were not used in the overall analysis). The starting voltage settings of the electrically stimulated sperm collection devices were all 3 V, with a maximum of 7 V and a minimum of 5 V; the shortest stimulation time was 3 minutes, and the longest was 9 minutes, with a wide range of individual variations; and the depth of insertion into the rectum, which was basically the same, was in the range of 3–7 cm (Table 2).

**Table 2:** Details of electroejaculation in Chinese pangolins (*n* = 5)

ID	Initial voltage (V)	Termination voltage (V)	Time (min)	Depth (cm)	Semen collection
Z3	3	6	8	3–5	Yes
Z7	3	5	3	3–6	Yes
Z11	3	7	9	3–6	Yes
Z15	3	5	5	3–5	Yes
Z2–1	3	6	5	3–7	Yes

**Table 3:** Individual and mean (standard error of the mean [SEM]) values of seminal characteristics of Chinese pangolin (*n* = 5)

ID	Volume (μL)	Concentration (10^6^/mL)	TMS (%)	PMS (%)	VSL (μm/s)	VCL (μm/s)	VAP (μm/s)	ALH (μm)	STR	LIN	BCF (HZ)	PMI (%)	ACR-I (%)
Z3	70	649.62	36.08	25.36	24.12	33.99	24.03	9.95	1.01	0.71	0.78	51.24	90.56
Z11	80	293.66	66.74	55.89	29.55	42.66	30.16	12.49	0.98	0.69	0.80	65.20	90.10
Z15	60	402.80	69.52	59.60	35.91	47.22	33.39	13.83	1.07	0.76	0.73	64.42	88.67
Z2–1	300	247.57	53.71	46.89	41.25	50.51	35.72	14.79	1.15	0.81	0.71	66.30	88.78
Mean	127.50	398.41	56.51	46.93	32.70	43.59	30.82	12.77	1.05	0.74	0.76	61.79	89.53
(SEM)	(57.64)	(89.84)	(7.63)	(7.67)	(3.72)	(3.58)	(2.53)	(1.05)	(0.04)	(0.03)	(0.02)	(3.54)	(0.47)
Z7(UC)	20	417.61	21.92	12.33	31.28	42.13	28.50	11.80	0.77	0.55	1.39	71.47	90.22

ACR-I: acrosome integrity; ALH: amplitude of lateral head displacement； BCF: beat/cross-frequency； LIN: linearity， PMI: plasma membrane integrity； PMS: progressively motile sperm； STR: straightness； TMS: total motile spermatozoa； UC: urine contaminated； VAP: average path velocity； VCL: curvilinear velocity；VSL: straight line velocity.

The semen of the Chinese pangolin was thicker and denser, with a yellowish colour ranging from pale yellow to dark yellow. The overall volume of ejaculate was low, and the volume of semen collected varied greatly between individuals (20–300 μL). The pH of the semen was alkaline, ranging from 7.7 to 7.9. The total volume of ejaculate was 127.50 ± 57.64 μL, the sperm concentration was 398.41 ± 89.84 × 10^6^/mL, the sperm viability was 56.51 ± 7.63%, the progressively motile sperm percentage was 46.93 ± 7.67%, the plasma membrane integrity was 61.79 ± 3.54% and the acrosome integrity was 89.53 ± 0.47. Among them, Z3 was lower than the other individuals in all indicators except semen concentration, which was higher than that of the other individuals, especially plasma membrane integrity (51.24%), which was much lower than the normal value indicator (60%) (Table 3).

**Table 4:** Values (mean ± SEM) for sperm morphometric evaluation in Chinese pangolins (*n* = 5)

ID	Head length (μm)	Head width (μm)	Tail length (μm)	Total length (μm)
Z3	10.44 ± 0.07^c^	1.23 ± 0.01^c^	54.65 ± 0.38^c^	65.09 ± 0.37^c^
Z7	10.81 ± 0.07^b^	1.24 ± 0.01^c^	55.06 ± 0.36^c^	65.88 ± 0.35^c^
Z11	10.62 ± 0.08^bc^	1.27 ± 0.01^b^	54.85 ± 0.36^c^	65.48 ± 0.35^c^
Z15	11.28 ± 0.09^a^	1.36 ± 0.01^a^	58.50 ± 0.32^a^	69.79 ± 0.32^a^
Z2–1	9.41 ± 0.08^d^	1.27 ± 0.01^b^	57.49 ± 0.33^b^	66.90 ± 0.33^b^
Mean	10.51	1.27	57.15	66.63
(SEM)	(0.04)	(0.005)	(0.20)	(0.16)

a,b,c,d Different lowercase letters show significant difference among males (*P* < 0.05).

**Table 5:** Values (mean ± SEM) for sperm morphometric evaluation in Chinese pangolins (*n* = 5)

ID	Head abnormality (%)	Tail abnormality (%)	Total (%)
Z3	16.25	11.67	28.33
Z7	9.91	21.12	31.03
Z11	9.97	11.55	24.93
Z15	10.9	8.05	18.96
Z2–1	20.14	10.07	30.94
Mean	13.43	12.49	26.84
(SEM)	(2.05)	(2.25)	(2.26)

### Morphometric evaluation

**Figure 2:**
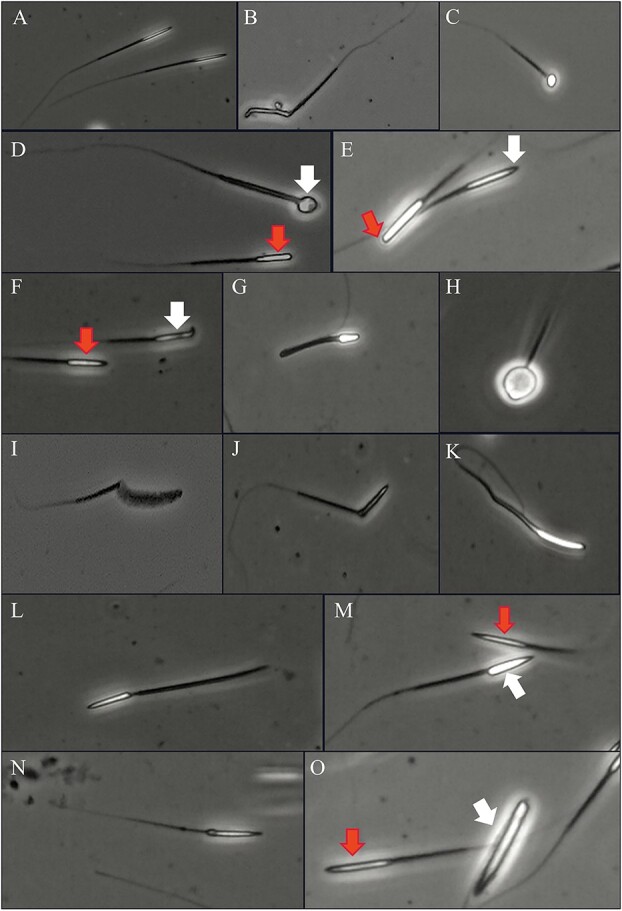
Spermatic morphology shown with phase contrast microscopy. (**A**) Normal sperm; (**B**) amorphous head; (**C**) spherical spermatozoa defect; (**D**) spherical sperm defects (white arrows) and acrosome detachment (red arrow); (**E**) acrosome detachment (red arrow) and normal sperm (red arrow); (**F**) sperm with hooked heads (white arrows) and normal sperm (red arrow); (**G**) micro head; (**H**) puffy head; (**I**) banana-shaped head; (**J**) proximal midpiece reflex; (**K**) head abnormality; (**L**) head-to-tail separation, thick middle piece; (**M**) head-to-tail translocation (white arrows) and normal sperm (red arrow); (**N**) proximal droplet; (**O**) swollen and narrow head (white arrow) and normal sperm (red arrow).

**Figure 3:**
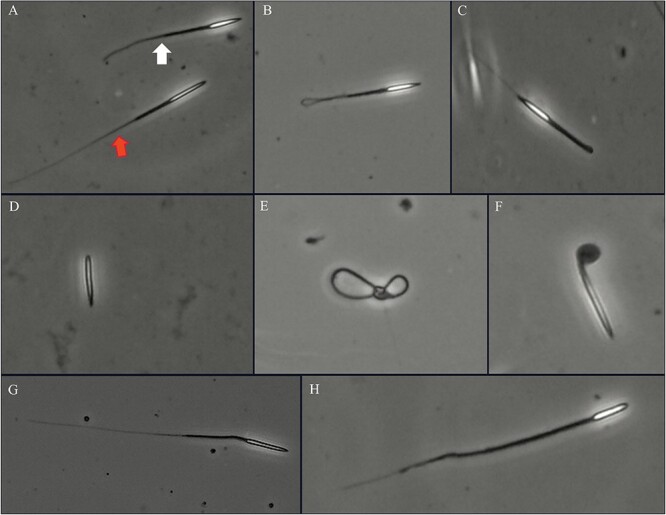
Spermatic morphology shown with phase contrast microscopy. (**A**) Short tail (white arrow) and normal spermatozoa; (**B**) coiled tail; (**C**) folded tail; (**D**) no tail; (**E**) bent tail; (**F**) very coiled tail; (**G**) bowed midpiece; (**H**) midpiece abnormality.

The head length, head width and tail length of pangolin spermatozoa were measured separately by image software. The head length was 10.51 ± 0.04 μm, the head width was 1.27 ± 0.005 μm, the tail length was 57.15 ± 0.20 μm and the total length was 66.63 ± 0.16 μm (see Table 4 for details). The average deformity rate of Chinese pangolin spermatozoa was 26.84 ± 2.26% [see Table 5 for individual and mean (SEM) values]. The most common morphological defects were head defects (13.43 ± 2.05%) (Fig. 2) and tail defects (12.49 ± 2.25%) (Fig. 3). Among them, common head defects included amorphous heads (Fig. 2B), spherical spermatozoa defects (Fig. 2C and D), acrosome detachment (Fig. 2D and E), head-to-tail translocation (Fig. 2M) and other defects; common tail defects included short tails (Fig. 3A), coiled tails (Fig. 3B and F), no tails (Fig. 3D) and other defects.

### Sperm micromorphology and ultrastructure

**Figure 4:**
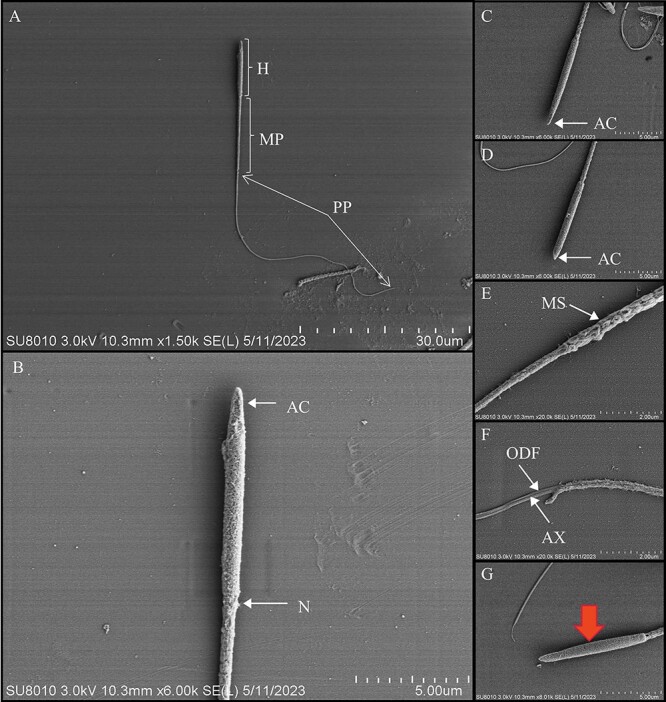
Scanning electron microscopy (SEM) photomicrographs of sperm morphology in Chinese pangolins. (**A**) H: head, MP: midpiece, PP: principal piece; scale bar 30 μm. (**B**) AC: acrosome, N: neck; scale bar 5 μm. (**D**) AC: acrosome; scale bar 5 μm. (**E**) MS: mitochondrial sheath; scale bar 2 μm. (**F**) AX: axoneme, ODF: outer dense fibres; scale bar 2 μm. (G) The head was fusiform (red arrow). Scale bar, 5 μm.

**Figure 5:**
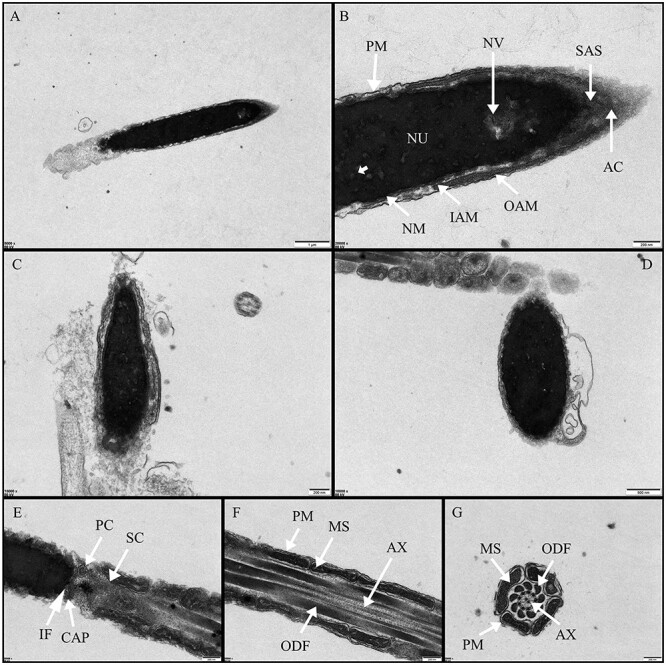
Transmission electron microscopy (TEM) photomicrographs of sperm morphology in Chinese pangolin. (**A**) Sperm head, scale bar 1 μm. (**B**) AC: acrosome, IAM: inner acrosomal membrane, NM: nuclear membrane, NU: nucleus, NV: nucleus, PM: plasma membrane, SAS: sub-acrosomal space. Scale bar 200 nm. (**C**) and (**D**) Sperm head cross-section and longitudinal section pictures, scale bar 200 and 500 nm. (**E**) CAP: capitulum, IF: implantation fossa, PC: proximal centriole, SC: segmented columns. Scale bar, 200 nm. (**F**) and (**G**) AX: axoneme, MS: mitochondrial sheath, ODF: outer dense fibres, PM: plasma membrane. Scale bar 200 nm.

From the scanning electron microscope (SEM) images of the spermatozoa of the Chinese pangolin, the head, neck, middle and tail of the spermatozoa could be clearly identified (Fig. 4A and B). The head of the spermatozoon was rod-shaped, distinct from the round or oval sperm head of most mammals. In addition, the overall paddle-shaped head of the spermatozoon of the Chinese pangolin was observed in the flat side view, with the anterior end of the acrosome nearly flattened and with obvious apical ridges (Fig. 4C and D). The middle part of the spermatozoon was encased by dense mitochondrial sheaths entangled with each other (Fig. 4E). In the scanned image of the longitudinal section of the sperm tail, axonemes and peripheral dense fibres could be observed (Fig. 4F), consistent with the results of transmission electron microscopy (Fig. 5D). In addition, abnormalities were observed in the head of the spermatozoa, which was enlarged in the middle and had an overall shuttle shape with an intact acrosome (Fig. 4G).

From the transmission electron microscopy images, it was clear that the sperm head of the Chinese pangolin consisted of a nucleus encapsulated by a nuclear membrane and an acrosome at the tip. The nucleus occupied most of the head (Fig. 5A), and the area of dense electron transmission spots was presumed to be a nuclear vesicle. The acrosome was enveloped by the intra- and extra-acrosomal membranes, and the dense layer at the posterior end of the acrosome lay between the plasma membrane and the nuclear membrane (Fig. 5B). Between the apical end of the sperm acrosome and the nucleus, a sub-acrosomal space was present (Fig. 5B). Notably, from the longitudinal and transverse sectioned transmission electron microscopy images of the sperm head, it was observed that the head of the spermatozoon had a multilayered membrane.

In this study, a longitudinal view of the neck (connecting the head and tail) of the Chinese pangolin spermatozoon was captured for the first time (Fig. 5E). The base of the nucleus was the forward-convex implantation fossa, which consisted of the capitulum (the implantation fossa was covered by a thick layer of dense material), proximal centriole and segmented columns. The periphery of the segmented columns was surrounded by mitochondria. The Chinese pangolin spermatozoon midpiece was composed of an axoneme, outer dense fibres and a mitochondrial sheath (Fig. 5F). The axoneme consisted of nine duplex microtubules and two central microtubules (Fig. 5G). This is similar to the composition of the sperm midpiece in most mammals. Observation of the longitudinal and transverse views of the spermatozoa’s midsection revealed that mitochondria were close to the outer dense fibres, helically entangled, with five in each loop, totalling approximately 50.

## Discussion

To our knowledge, this is the first study to collect and evaluate the semen of the Chinese pangolin, as well as the first to systematically describe the morphology of Chinese pangolin spermatozoa. It provides valuable experience and a foundation for follow-up studies of semen-related research in the Chinese pangolin.

Because the Chinese pangolin is timid by nature and curls up when disturbed, some natural methods of semen collection, such as an artificial vagina (AV), have been abandoned (Nadaf *et al.*, 2022). In this study, several common methods of semen collection were tried in a pre-experiment: the rectal massage method, electroejaculation method and hand collection method (Palmer *et al.*, 2005; Jimenez-Rabadan *et al.*, 2016; Moore and Hasler, 2017; Tarmizi *et al.*, 2020b); the electroejaculation method was found to have the highest success rate. Researchers have also successfully collected semen using electroejaculation on Malay pangolin (Tarmizi *et al.*, 2020a), as well as endangered mammals that share the same diet as pangolins, such as the six-banded armadillo (*Euphractus sexcinctus*) and the giant anteater (*Myrmecophaga tridactyla*) (Serafim *et al.*, 2010; Sousa *et al.*, 2013; Luba Cdo *et al.*, 2015). During semen collection, it was found that the Chinese pangolin has a multisegmented ejaculate. In addition, the Chinese pangolin has a low semen volume and ejaculates in a gushing manner. In the artificial collection of semen in other species, a drug-assisted method is generally adopted to promote and induce ejaculation in animals. This method is heavily utilized in domestic animals (cats, donkeys) as well as wild animals (white rhinoceros) with good results (Silinski *et al.*, 2002; Madrigal-Valverde *et al.*, 2020; Khanam *et al.*, 2021). Therefore, the administration of alpha 2-adrenergic agonists to the Chinese pangolin as an ejaculatory inducer and facilitator prior to sperm collection may be considered.

During semen collection, the phenomenon of multiple ejaculations in small amounts over a short period of time occurred after the initial signs of ejaculation. In the video-screen recordings of captive Chinese pangolins mating accumulated by our research team over a long period of time, we observed the phenomenon of multiple mating in Chinese pangolins, which lasted for approximately a week, similar to some felids such as Amur tigers (*Panthera tigris altaica*) and leopards (*Panthera pardus*) (Owen *et al.*, 2010; Gu *et al.*, 2016). The Malayan pangolin, which is in the same family, exhibits the same reproductive behaviour in long-term captive breeding (Yan *et al.*, 2023b). Therefore, we surmised that the ejaculation mode of male Chinese pangolin may be a small amount in multiple segments. In addition, Chinese pangolins can mate throughout the year under captive conditions, and this mating pattern also exists in Malayan pangolin (Yan *et al.*, 2023a). We speculate that the ovulation and reproductive cycle of female Chinese pangolins may be closely related to the ejaculation mode and mating pattern of male Chinese pangolins, and further studies are needed in this area.

There are no relevant reports on the semen concentration of the Chinese pangolin, and this study found that the semen concentration varied greatly among individuals, with a maximum concentration of 649.62 × 10^6^/mL and a minimum concentration of 247.57 × 10^6^/mL. However, the semen concentration of 15 Malayan pangolins (997.19 ± 728.98 × 10^6^/mL) was determined by researchers (Tarmizi *et al.*, 2020b). Compared to the Malayan pangolin, the semen concentration of the Chinese pangolin was slightly lower but still higher than that of other mammals (Silva *et al.*, 2015; Carvalho *et al.*, 2020). Because the species ejaculates small amounts of semen, high concentrations of semen may be a strategy for pangolins to increase conception rates.

The pH of semen plays a critical role in semen quality (Contri *et al.*, 2013). In addition, the storage of semen requires a suitable environmental pH value, and the accuracy of semen pH is one of the key factors affecting the formulation of semen extenders (Liu *et al.*, 2016). Therefore, accurate semen pH data are crucial for further research and storage of Chinese pangolin semen. In this study, the semen pH values of Chinese pangolin were all within the range of 7.7–7.9, which is an alkaline range. It was hypothesized that these results might be caused by the use of electrical stimulation, because some studies have shown that electrical stimulation of semen collection leads to an increase in the pH of the expelled semen by stimulating the parasympathetic glands (Dooley and Pineda, 1986; Setchell *et al.*).

Sperm morphometry is an important component of sperm morphology for assessing variations and differences in size between different regions of the cell and can help to determine sperm size standards between different species (Beracochea *et al.*, 2014). The measurements of the Chinese pangolin spermatozoa in this study were similar to previous measurements of the spermatozoa of the Taiwanese subspecies of the Chinese pangolin from the epididymis (Chang *et al.*, 2020), with similar values for head length and head width and only slightly higher values for tail length and total length, suggesting that the spermatozoa morphology measurements of the Chinese pangolin in the present study have a high degree of confidence.

In the present study, a high percentage of normal spermatozoa (73.16 ± 2.26%) was observed, which is consistent with the range of 70%–80% for the normal percentage of spermatozoa in semen (Kuster *et al.*, 2004). Notably, an interesting type, spherical spermatozoa, was found when observing malformed spermatozoa (Fig. 2C and D). This malformation was also observed in spermatozoa obtained from the male epididymis of the Taiwanese subspecies of the Chinese pangolin (Chang *et al.*, 2020). This is a very rare type of sperm malformation (Modarres *et al.*, 2016; Zhi *et al.*, 2016), especially among rod-shaped spermatozoa. However, the study of spherical spermatozoa is relatively rare in animals and has been studied more in human spermatozoa. This type of aberration is known as globozoospermia, and the main mechanisms of formation include acrosomal malformation or loss of the acrosome (Han *et al.*, 2017) and gene deletion (Lu *et al.*, 2006; Liu *et al.*, 2010; Elinati *et al.*, 2012). In the staining test for acrosome integrity and morphological observation, some spherical spermatozoa with missing acrosomes were observed. Therefore, it can be inferred that acrosome deletion may be one of the reasons for the formation of spherical spermatozoa in the Chinese pangolin. Further studies are needed to confirm the cause at the genetic level. In addition, spherical spermatozoa can seriously affect the conception rate or even cause infertility or the formation of malformed foetuses (Dam *et al.*, 2007), and the effect of this in the reproduction process of pangolins remains to be further determined.

Scanning electron microscopy analysis showed that Chinese pangolin spermatozoa have a rod-shaped head and a long tail. Surprisingly, this is quite different from most mammals; instead, it is closer to the sperm shape of avians (e.g. penguins) and reptiles (e.g. iguanas) (Vieira *et al.*, 2004; Mafunda *et al.*, 2017; Kustra *et al.*, 2019). It has been demonstrated that this head shape may be able to obtain a higher forward speed; also, a higher sperm head aspect ratio increases the competitiveness of the spermatozoa (Tourmente *et al.*, 2011; Ramon *et al.*, 2013). Compared with the round and oval heads of mammals in general, the Chinese pangolin has a relatively high head aspect ratio, which may be conducive to improving the competitiveness of spermatozoa.

In analysing the transmission electron microscopy images of the spermatozoa of the Chinese pangolin, we obtained a result that was quite different from that of our predecessors. By analysing transmission electron microscopy images of spermatozoa obtained from male epididymal dissections of the Taiwanese subspecies of the Chinese pangolin, Chang *et al.* (2020) concluded that the acrosomal membrane does not have a double membrane structure (outer and inner) as in rodents or other mammals (Tsai *et al.*, 2012) but rather a multiple bi-lamellar membrane structure. On the contrary, the present study clearly observed the inner and outer double membranes of the acrosome (Fig. 5B). We surmised that the reason for this discrepancy is because Chang *et al.* (2020) used sperm from the epididymis. As widely recognized, there are sperm at various developmental stages in the epididymis (Cosentino and Cockett, 1986; Jankovicova *et al.*, 2018).

In this study, we present the first transmission electron microscopy images of the sperm neck structure of the Chinese pangolin, which was found to be similar to that of most mammalian sperm necks. The entire neck is funnel-shaped, with a cylindrical proximal centriole located in the anterior portion of the neck, and the posterior portion of the neck is connected to the middle portion of the spermatozoa tail. The segmented columns have peripheral mitochondrial sheaths that are not yet formed, and mature mitochondrial sheaths are formed only in the portion that connects to the middle section. At the same time, we observed that the degree of forward concavity of the implantation fossa at the posterior end of the sperm nucleus is not large, unlike in most mammals. It has been demonstrated that a strong sperm head-tail coupling apparatus (HTCA) is required to ensure that the spermatozoa maintain an intact morphology on their way to converge with the egg, and this device is the implantation fossa, which is used to strengthen the attachment of the tail to the head (Wu *et al.*, 2020a). However, it is unclear whether this shallower implantation fossa of the Chinese pangolin has unique reproductive significance.

The midsegment of the Chinese pangolin spermatozoon tail is similar in structure to that of other mammals (Gu *et al.*, 2019). The periphery of the midsegment is a mitochondrial sheath arranged in a helical tangle, which produces adenosine triphosphate (ATP), supplying energy for the flagellar motility of the spermatozoon (Park and Pang, 2021). The Chinese pangolin has approximately 50 spirochetes in the midsegment, which is closer to the number in some mammalian animals, such as 48 in the impala (*Aepyceros melampus*), 54 in the jaguar (*Panthera onca*) and 45 in the six-banded armadillo (Ackerman *et al.*, 1999; Sousa *et al.*, 2013; Silva *et al.*, 2019). However, the number of mitochondria directly affects energy production and, thus, fertilization (Anderson and Dixson, 2002). Therefore, more studies are needed to link this quantitative trait to certain reproductive characteristics and advantages of the species.

Overall, this study opens up a feasible path for the continuation of research on pangolin spermatozoa, cryopreservation of semen, the establishment of a gene bank and the development of assisted reproductive technology. It also provides a theoretical basis for conservation studies in Chinese pangolins and ultimately in all members of the order Pholidota.

## Conclusion

In this study, the semen of the Chinese pangolin was successfully collected using the electroejaculation method. We further established baseline values for semen quality assessment indices. The observation of sperm ultrastructure found that the spermatozoa of the Chinese pangolin was specialized rod-shaped, which was different from that of most mammals. We also found that the acrosome membrane of the Chinese pangolin was a double membrane structure rather than a multiple bi-lamellar membrane structure as reported by a previous study. Therefore, this study provides technical support for continued research with sperm cryopreservation, gene bank establishment and population restoration using assisted reproductive technologies. Further, it lays a practical foundation for implementing conservation programmes in Manidae family animals.

## Author Contributions

K.W. designed the experiment; Y.L., X.X., X.W., F.A. and Z.R. carried out the animal experiment and sample collection; Z.X., Y.H. and Y.L. performed the statistical analyses; S.Z. processed the image; Y.L. and K.W. wrote the manuscript. All authors read and approved the final manuscript.

## Conflicts of Interest

The authors declare no conflict of interest.

## Funding

This study was funded by the National Key Program of Research and Development, Ministry of Science and Technology (No. 2022YFF1301500).

## Data Availability

All underlying data are incorporated in the article.
